# Influence of Spirituality on Cool Down Reactions, Work Engagement, and Life Satisfaction in Anthroposophic Health Care Professionals

**DOI:** 10.1155/2015/754814

**Published:** 2015-01-28

**Authors:** Arndt Büssing, Désirée Lötzke, Michaela Glöckler, Peter Heusser

**Affiliations:** ^1^Quality of Life, Spirituality and Coping, Institute of Integrative Medicine, University of Witten/Herdecke, Gerhard-Kienle-Weg 4, 58313 Herdecke, Germany; ^2^Medical Section of the School of Spiritual Science at the Goetheanum, Dornach, Switzerland; ^3^Chair for Theory of Medicine, Integrative and Anthroposophic Medicine, Institute of Integrative Medicine, Witten/Herdecke University, Herdecke, Germany

## Abstract

This study aimed to analyse whether spirituality is a resource for health care professionals to deal with increasing stress and work burden, specifically to analyse associations between “cool down reactions” (which describe an emotional distancing towards patients and/or reduced engagement as a strategy to protect their own functionality), work burden, and life satisfaction. We specifically focussed on anthroposophic health care professionals because of their unique approach to distinct aspects of spirituality. In a cross-sectional survey using standardized questionnaires, 489 persons were enrolled (66% women, mean age 53 ± 10 years, 41% physicians, 12% nurses, and 47% other health care professionals). They scored very high on all measures of spirituality and moderate to low with respect to “cool down reactions.” Significant predictors of “cool down reactions” were low work vigor, perceived work burden, alcohol consumption, low life satisfaction, and *religious orientation* (*R* ^2^ = 0.20). In contrast, their life satisfaction was explained best (*R* ^2^ = 0.35) by vigor, with further positive influences of being a physician, *conscious interactions*, and living with a partner on one hand and negative influences of “cool down reactions,” work burden, and *transcendence convictions* on the other hand. Thus, specific aspects of spirituality have only a small influence on anthroposophic health care professionals' “cool down reactions,” but might buffer against a loss of vigor and dedication in their work.

## 1. Introduction

Due to different reasons, in the past decades, the stress potential for health care professionals increased [1–3]. Structural changes in the health care system (i.e., implementation of the health-related-diagnosis-groups system) led to a rise of pressure to reduce health care costs and hospital stays, work intensification, increasing administrative tasks, and increasing heteronomy. In fact, health care professionals have to deal “with the most emotionally distressing of situations-illness, dying, suffering in every form” [4]. Humphries et al. [5] summarized further factors like heavy workloads, staffing shortages, or high staff turnover rates, which contribute to higher stress levels.

Health care professionals are exposed to a large psychological burden, too [6–8]. Their increasing work burden is mostly associated with poor psychological wellbeing, emotional exhaustion, reduced life satisfaction, dissatisfaction with the job and their own quality of work, and so forth or even with their decision to quit the job [9–12]. Several of these symptoms are characterized as “burnout” as described by Maslach et al. [13], that is, emotional exhaustion, depersonalisation, and reduced personal accomplishment.

It is obvious that health care professionals have to face the implications of work stress and to find strategies to adapt and to stay “functional” while doing their job. Using the term “cool down reactions,” we will summarize (and measure) experiences of emotional exhaustion and emotional distancing from the patients to keep “functionality” in the job. Different strategies might be chosen to cope with the adverse working conditions and to protect oneself from burnout syndrome. Common strategies are an emotional distance from the suffering person or cynic reactions [14, 15] which can be categorized as “depersonalization” [15]. Interestingly, McManus et al. [14] found that doctors' cynical reactions may reduce their stress.

Although it is not a desired reaction with respect to an adequate health care and support of patients, this “depersonalization” might nevertheless be an adaptive strategy for health care professionals. Emotional distance from the patients is implicitly regarded by most health professionals as a common reaction of self-protection. This “professional neutrality” is kept even when facing difficult medical situations and appalling fate of patients, to protect their own “functionality” in the job.

While it is evident that for patients their spirituality is a resource to cope with chronic diseases (summarized in [16, 17]), there are accumulating data that spirituality can be also a resource to cope for physicians. According to the results of Zwack and Schweitzer [18] based on data of German physicians, spirituality is one of several resilience strategies to deal with job-related stress factors. Also, Anandarajah and Roseman [19] stated that “spiritual self-care […] could contribute to improved compassionate patient care and help “immunize” physicians against burnout.” Doolittle et al. [20] concluded that they “identified […] spiritual coping strategies that may have protective benefit” for physicians. They said that “along with other coping techniques, spirituality may be an important factor in mitigating the effects of burnout.”

In a large sample of US physicians, half of them stated that they “try hard” to carry their religious beliefs over into all their “other dealings in life” and that they “look to God for strength, support, and guidance” [21]. Among paediatric oncologists from the US, half of them assumed that their spiritual/religious beliefs influence the interactions with patients and colleagues [22]. A smaller study enrolling 53 US surgeons found that their “religious beliefs play a part” in their clinical practice, that they feel “guided by a higher power than my own abilities” in their practice, and that their religious beliefs were helpful to deal with their “inability to heal patients with poor prognoses” [23]. In a sample of German psychiatrists, 54% stated that “Religious beliefs influence whole approach to life,” 37% of them tried to “carry religion into other aspects of life,” and also 36% “experience God's presence” [24]. Thus, physicians' spirituality seems to be of importance for their own life and might be a resource to cope with difficult situations, too.

Because health care professionals deal with increasing adverse working conditions, which may affect their empathic encounter with patients and their life satisfaction, we aimed to analyse whether spirituality is a resource for health care professionals to cope with increasing stress and work burden, specifically with respect to “cool down reactions” which describe an emotional distancing and development of cynic reactions towards patients and/or reduced engagement as a strategy to protect their own functionality. For this analysis, we focussed on health care professionals with an anthroposophic background because of their unique approach to distinct forms of spirituality.

Anthroposophy (an empirical form of “spiritual science”) is a humanistically oriented spiritual philosophy developed by the Austrian Philosopher Rudolf Steiner (1861–1925). It is based on a holistic anthropology according to which the human being cannot solely be explained by the molecules of the physical body and their interactions, but more comprehensively by holistically organized interactions of physical and nonphysical or “spiritual” factors: body, life, soul, and spirit [25]. In the anthroposophic theory of medicine and the practice of health care, this has led to an extension of conventional care and to integrative medical concepts, using a multimodal treatment approach, in the attempt to account for physical as well as spiritual factors in the understanding of health and disease and treatment of patients [26]. Apart from spiritual factors in humans and nature, anthroposophy postulates the existence of an objective spiritual world, potentially accessible to specifically trained direct experience, that is, by perceptive imagination, inspiration, and intuition [27]. The specific training consists of systematic enhancement of inner awareness by meditation and cultivating one's capacity of thinking, feeling, and moral conduct [28, 29]. This “spiritual path” intends to lead to refined perception, insight, judgment, and decision. In anthroposophic health care professions, personality development and an inner spiritual path are described as inherent parts of self-training and professionalism which may have an influence on diagnosis finding, treatment ideas, and empathic support of patients [27–33].

## 2. Methods

### 2.1. Participants

All individuals of this anonymously conducted cross-sectional study were invited by the head of the Medical Section of the Goetheanum, Dr. Michaela Glöckler, via email using the respective network of associated professional associations. The Medical Section of the School of Spiritual Science at the Goetheanum coordinates and supports research and is the head organization of anthroposophic medicine. For this project, the Medical Section contacted associated health care professionals to fill the questionnaire. Participants received the respective German language questionnaire anonymously either as a hard copy via surface mail or as an electronic document via email. To account for the fact that any analysis enrolling persons with a wide spectrum of different professions has to deal with differences in specific world view and specific views related to the respective profession, we chose to focus on health professions within the field of anthroposophic medicine which (should) share a similar philosophical background.

For this analysis, we only enrolled persons with health related professions (*n* = 489; i.e., medical doctors, nurses, therapists, etc.), while all other respondents or those with unclear professions were excluded from the sample (*n* = 105). Sociodemographic data and occupational tasks of respondents were given in Table 1.

## 3. Measures

### 3.1. Cool Down Index

We intended to operationalize and measure the aforementioned “cool down reactions” as phases of emotional exhaustion and distancing from the patients to protect the own “functionality,” using the 9-item cool down index (CDI), designed by Arndt Büssing (Table 2) [34]. These 9 items address the perception of emotional exhaustion when caring for the patients (i.e., CDI 3—their personal problems and worries often simply become too much for me; CDI 8—I myself increasingly go short; CDI 6—I increasingly think how nice it would be to pack it all in; CDI 7—some of them simply annoy me), subsequent reactions of emotional withdrawal (CDI 2—I have to withdraw with increasing frequency to protect myself; CDI 1—I simply must stop letting everything get to me to such an extent; CDI 4—I often no longer have the patience to listen to them; CDI 5—I largely don't care what they think of me), and “working to rule” as a strategy (CDI 9—I increasingly “work to rule”). All items were introduced by the sentence “In dealing with the people I look after (therapeutically), I notice that….”

Respondents have to judge how often they perceive the respective feelings (1—a few times a year or less; 2—once a month or less; 3—a few times a month; 4—once a week; 5—a few times a week; 6—every day) and the strength of these feelings and perceptions with scores ranging from 1 (weak) to 6 (very strong). The sum of both scores indicates the significance of the respective feeling on a single item level (scores may range from 2 to 12), while the sum of all 9 items constitutes the CDI mean score.

These “cool down reactions” can, but must not necessarily, be associated with clinically relevant burnout symptoms. First findings with the CDI among a sample of nurses from Austria found a strong correlation between burnout symptoms (as measured with the Maslach Burnout Inventory) and the CDI (*r* = .65; *P* < .0001) [34].

### 3.2. Work Engagement

The Utrecht Work Engagement Scale (UWES) measures “a positive, fulfilling, work-related state of mind that is characterized by vigor, dedication, and absorption” [35]. For this study, we used the 9-item shortened version (UWES-9; alpha ranging between .85 and .92) which has similar psychometric properties to the long version. It has been shown that work engagement is negatively associated with burnout [36] and positively related to work and life satisfaction and self-rated health [35].

Specific items are “I am enthusiastic about my job,” “At my work, I feel bursting with energy,” “At my job, I feel strong and vigorous,” “I am proud of the work that I do,” “I am immersed in my work,” and so forth. The items are scored on a 7-point Likert scale, ranging from “never” to “always/every day.”

The UWES differentiates work engagement with three subdimensions, that is, vigor, dedication, and absorption.

### 3.3. Life Satisfaction

Life satisfaction was measured using the Brief Multidimensional Life Satisfaction Scale (BMLSS; alpha = .87) [37]. The items address intrinsic (myself and life in general), social (friendships and family life), external (work situation and where I live), and prospective dimensions (financial situation and future prospects) of life satisfaction and also satisfaction with the abilities to manage daily life concerns and satisfaction with the health situation.

Each of these 10 items was introduced by the phrase “I would describe my level of satisfaction as…,” and scored on a 7-point scale ranging from dissatisfaction to satisfaction (0—terrible; 1—unhappy; 2—mostly dissatisfied; 3—mixed (about equally satisfied and dissatisfied); 4—mostly satisfied; 5—pleased; 6—delighted). The BMLSS sum scores were referred to as 100% level (“delighted”). Scores >50% indicate higher life satisfaction, while scores <50% indicate dissatisfaction.

### 3.4. Self-Perceived Work Burden

Current work burden was measured with a visual analogue scale (VAS) ranging from 0 (no work burden) to 100 (unbearable work burden).

### 3.5. Aspects of Spirituality

To quantify cognitive, emotional, intentional, and concrete activities of theism/belief, pantheism/transcendence, existentialism, and humanism, we used the ASP (aspects of spirituality) questionnaire [38]. The 25-item instrument has a very good internal consistency (Cronbach's alpha = .94) and differentiates 4 main factors which would explain 65% of variance [39], that is,

1. *religious orientation: prayer/trust in God* (9 items; alpha = .93; religious views),
2. *search for insight/wisdom* (7 items; alpha = .88; philosophical/existential views),
3. *conscious interactions* (5 items; alpha = .83; relational views),
4. *transcendence conviction* (4 items; alpha = .85; transcendent spiritual views).

All items were scored on a 5-point scale from disagreement to agreement (0—does not apply at all; 1—does not truly apply; 2—does not know (neither yes nor no); 3—applies quite a bit; 4—applies very much). The mean scores were referred to as 100% level (4 “applied very much” = 100%). Scores >60 would indicate a high relevance of the respective aspect of spirituality for the individuals, while scores <40 would indicate a low relevance; scores between 40 and 60 would indicate an indifferent attitude.

### 3.6. Statistical Analyses

Descriptive statistics, internal consistency (Cronbach's coefficient *α*), and factor analyses (principal component analysis using varimax rotation with Kaiser's normalization), as well as analyses of variance, first order correlations, and regression analyses, were computed with SPSS 22.0.

Given the exploratory character of this study, significance level was set at *P* < .05.

With respect to classifying the strength of the observed correlations, we regarded *r* > .5 as a strong correlation, an *r* between .3 and .5 as a moderate correlation, an *r* between .2 and .3 as a weak correlation, and *r* < .2 as no or a negligible correlation.

## 4. Results

### 4.1. Participants

Among the 489 enrolled health care professionals with a mean age of 53 ± 10 years, 66% were women and 34% men (Table 1). Most were living with a partner and were affiliated to the Christian Community (‘Christengemeinschaft'). Within the sample, 41% were physicians, 12% nurses, 19% art/music therapists, 14% eurythmy therapists, and 14% other health care professionals.

Health care professionals' perceived work burden was moderately high (mean scores 53.0 ± 22.8), while their general life satisfaction was high, too (mean scores 76.3 ± 12.5). All aspects of spirituality scored high to very high, particularly* transcendence conviction* (Table 1).

All further details are presented in Table 1.

### 4.2. Cool Down Reactions

As shown in Table 2, the 9 items of the CDI showed a good internal consistency (Cronbach's alpha = .840) in this sample. Here, the mean CDI score was 36.3 ± 14.2. Although work engagement and perceived work burden differed significantly with respect to gender, age, educational level, and profession, these variables had no significant influence on CDI (Table 3).

Using the CDI cut-off scores from a reference sample of hospital physicians (Recchia et al., in preparation), 77% of the sample scored low (CDI < 46.0), 12% moderate (CDI between 46.0 and 55.5), and 11% high (CDI > 55.5) with respect to “cool down reactions.” Also these categorized CDI scores did not significantly differ between physicians and other health care professionals (data not shown).

### 4.3. Correlation Analyses

Because we intended to analyse whether different aspects of spirituality were associated with work burden, cool down reactions, and life satisfaction, we performed first order correlation analyses.

As shown in Table 4, the CDI scores correlated moderately (and negatively) with life satisfaction and the vigor component of work satisfaction. Among the specific aspects of spirituality, only* conscious interactions* correlated significantly, yet marginally negatively, with the CDI.

Perceived work burden correlated weakly negatively with life satisfaction, and only marginally (and negatively) with* conscious interactions*. In line with this, work engagement correlated moderately (positively) with life satisfaction and also moderately with* religious orientation* and* search for insight/wisdom* and weakly positively with* conscious interactions* and* transcendence convictions*.

Life satisfaction was moderately associated with work engagement and weakly associated with* conscious interactions*.

### 4.4. Predictors of Cool Down Reactions and Life Satisfaction

Because many variables may have an impact on health care professionals' “cool down reactions” on one hand and their life satisfaction on the other hand, we performed stepwise regression analyses to identify significant predictors. The following variables were included in the respective prediction models: gender, age, high school education, living with or without a partner, physicians versus other professionals, duration of years working on the respective profession, work engagement, perceived work burden, alcohol consumption, and aspects of spirituality.

As shown in Table 5, “cool down reactions” can be explained best by perceived work burden, with further negative influences of the work vigor component and life satisfaction and positive influences of alcohol consumption and* religious orientation*. These variables explain 20% of variance.

In contrast, health care professionals' life satisfaction was explained best by vigor, with further positive influences of being physicians,* conscious interactions*, and living with a partner on one hand and negative influences of “cool down reactions,” perceived work burden, and, however,* transcendence convictions* on the other hand (Table 5). These variables explain 35% of variance. All aspects of spirituality explain only 6% of variance in life satisfaction, with* conscious interactions* being the only significant variable (beta = .21; *T* = 3.9; *P* < .0001).

## 5. Discussion

The aim of the study was to investigate whether spirituality is a resource for health care professionals with an anthroposophic background to cope with increasing stress and work burden, specifically with respect to “cool down reactions.” Due to their specific and unique philosophical and spiritual convictions (encouraging personality development and an inner spiritual path which may also have influence on empathic support of patients [27–33]), the enrolled health care professionals scored high to very high on all scales of the ASP questionnaire, particularly on* transcendence conviction* and* search for insight/wisdom*. Also, in a previous study, we have found that physicians with a specialisation in complementary and/or alternative medicine or anthroposophic medicine had significant higher scores on these aspects of spirituality when compared to “conventional” physicians lacking such specialisations [40]. We can thus confirm that specific aspects of spirituality are in fact of strong (self-assessed) importance to anthroposophic health care professionals. Yet it is unclear whether or not these aspects of spirituality are related to their work burden, “cool down reactions,” and finally life satisfaction.

We found that with respect to their perceived work burden, the scores were moderately high, while their life satisfaction was high. In line with this, they scored moderate to low on the CDI. The mean score of the CDI of the whole sample was 36.3 (SD = 14.2) and that of physicians was 37.6 (SFD = 15.2), thus, lower than the score of a reference sample of 467 hospital physicians (mean CDI = 47.8; SD = 18.10) (Recchia, Falkenberg et al. in preparation). This might indicate that either health care professionals (and specifically physicians, too) with an anthroposophic background (and its specific implications for their daily life activities) may have access to strategies (i.e., specific spiritual practices) helpful to cope with stressful working conditions and thus avoiding cool down reactions. Alternatively it might be that their working conditions are not as stressful as hospital physicians. Although we have no specific data to verify this, we would assume that a large fraction of the enrolled physicians of this sample are working as practitioners rather than as hospital doctors, and thus the stress conditions might be different. Moreover, studies with other health care professionals (i.e., nurses and psychotherapist) are currently underway. So far, we do not assume that our data can be easily transferred to health care professionals in general, because the specific context of anthroposophy might be crucial. Further studies are needed.

Nevertheless, our results show that the influence of spirituality on “cool down reactions” is much smaller than expected, because the different aspects of spirituality were not indicated as significant predictors of “cool down reactions,” and there was only a marginal negative correlation for* conscious interactions*. Instead, the potential of spirituality for physicians, nurses, and other health care professionals dealing with stressful working conditions can be possibly seen in positive correlation on their work engagement. In this study, all specific aspects of spirituality correlated weakly to moderately positively with the work engagement and weakly with life satisfaction (which are both moderately interconnected). This can be seen in line with findings of Zwack and Schweitzer [18] that spirituality is one of several resilience strategies to deal with job-related stress factors or that spirituality “could contribute to improved compassionate patient care” [19] and might be beneficial factor to ameliorate or prevent burnout symptoms [19, 20]. Using qualitative interviews, Anandarajah and Roseman [19] substantiated that the interviewed physicians, “despite diversity of personal spiritual beliefs,” felt that compassion, as one aspect of spirituality, was “essential for a physician.” In our study, the variable compassion is part of the* conscious interactions* subscale, which is weakly associated with work engagement and life satisfaction and the only aspect of spirituality which is related to lower cool down scores at all. Among resident physicians, Doolittle et al. [20] found that physicians “who place a high priority on healthful relationships, engage in an active spiritual life, and practice humility [*sic*] may have important personality traits that protect against burnout.” All these aspects refer to relational aspects of spirituality, which are part of the “spiritual path” of persons with an anthroposophic background, too.

Although our results give a weak hint that particularly the relational forms of spirituality (i.e.,* conscious interactions*) might be a resource to cope with stress in working life, the study design does not allow causal conclusions. Further studies with other samples of health care professionals are required, because one cannot exclude the possibility of too positively assessed aspects of spirituality (in terms of desired or even realized attitudes and behaviours) in this unique sample of persons with an anthroposophic worldview.

In conclusion, health care professionals with an anthroposophic background scored very high on all aspects of spirituality and relatively low on the CDI scores (which would indicate relatively low emotional exhaustion and low distancing from their patients). Their spirituality does not necessarily contribute to their general life satisfaction but might be a buffer against a loss of vigor and dedication in their work.

## Figures and Tables

**Table 1:** Characteristics of 489 health professionals.

Variables	Mean/%
Gender, %	
Women	66
Men	34
Age, mean, standard deviation (years)	53.3 ± 10.2
Family status, %	
Living with partner—married	56
Living with partner—not married	14
Divorced	14
Single	17
Educational level, %	
Secondary school (Hauptschule)	3
Junior high school (Realschule)	11
High school (Gymnasium)	62
Others	24
Profession, %	
Physicians	41
Nurses	12
Art/music therapists	19
Eurythmy therapists	14
Others	14
Denomination, %	
Christian community	44
Catholic	11
Protestant	13
Others	2
None	31
Aspects of spirituality (ASP; 0–100)	
*Religious orientation *	78.0 ± 17.7
*Search for insight/wisdom *	88.3 ± 12.8
*Conscious interactions *	82.2 ± 11.4
*Transcendence conviction *	95.8 ± 11.7
Duration of years working in the profession (years)	21.6 ± 11.5
Perceives work burden (VAS; 0–100)	53.0 ± 22.8
Life satisfaction (BMLSS; 0–100)	76.3 ± 12.5
Alcohol consumption (%)	
Never	47
1x per month	23
2-3x per month	14
1-2x per week	11
Several times per week	6

**Table 2:** Mean values and reliability analysis of CDI's single items Cool.

CDI single items (with identifying item numbers)	Mean ± SD	Corrected item—scale correlation	*α* if item is deleted (*α* = .840)
CDI 1—I simply must stop letting everything get to me to such an extent	5.5 ± 2.8	.498	.831
CDI 2—I have to withdraw with increasing frequency to protect myself	5.1 ± 2.7	.615	.816
CDI 3—their personal problems and worries often simply become too much for me	4.0 ± 2.3	.637	.815
CDI 4—I often no longer have the patience to listen to them	4.0 ± 2.3	.646	.813
CDI 5—I largely don't care what they think of me	3.8 ± 2.5	.418	.838
CDI 7—I increasingly think how nice it would be to pack it all in	3.1 ± 2.0	.561	.824
CDI 8—some of them simply annoy me	3.7 ± 2.2	.565	.822
CDI 9—I myself increasingly go short	4.0 ± 2.6	.552	.824
CDI 10—I increasingly “work to rule”	3.2 ± 2.0	.518	.827

**Table 3:** Mean values within the sample.

		Cool down index	Work engagement	Perceived work burden	Life satisfaction
All individuals	Mean	36.3	4.0	53.0	76.3
	SD	14.2	1.0	22.8	12.5

Gender					
Women	Mean	36.44	4.10	51.22	75.67
	SD	14.37	1.02	22.03	12.33
Men	Mean	36.28	3.87	56.48	77.92
	SD	13.97	.96	23.57	12.37

*F* value		.0	**6.0**	**5.8**	3.6
*P* value		n.s.	**.015**	**.016**	.059

Age					
31–40 years	Mean	39.85	3.68	53.31	77.06
	SD	14.26	1.02	21.32	13.06
41–50 years	Mean	35.69	3.97	53.94	76.94
	SD	12.49	.97	20.24	11.59
51–60 years	Mean	36.90	4.00	54.64	74.54
	SD	15.11	1.02	21.65	12.62
61–70 years	Mean	34.08	4.32	47.40	79.37
	SD	14.50	.98	25.93	11.89

*F* value		2.0	**5.1**	2.4	**3.4**
*P* value		n.s.	**.002**	.066	**.018**

Profession					
Medical doctors	Mean	37.57	3.78	54.43	79.49
	SD	15.21	1.01	23.39	11.86
Nurses	Mean	37.75	3.78	51.81	76.16
	SD	14.20	1.20	21.80	13.60
Art/music therapists	Mean	34.20	4.27	47.23	72.09
	SD	12.82	.90	22.14	12.83
Eurythmy therapists	Mean	35.28	4.32	57.90	71.66
	SD	14.16	.95	21.45	11.45
Other	Mean	35.11	4.23	52.58	77.51
	SD	12.76	.86	23.33	11.39

*F* value		1.2	**7.7**	**2.6**	**8.9**
*P* value		n.s.	<**.0001**	**.039**	**<.0001**

Education level					
High school education	Mean	37.13	3.91	55.36	76.41
	SD	14.25	1.01	21.91	13.01
Other levels	Mean	34.94	4.18	49.21	76.03
	SD	14.06	1.00	24.00	11.81

*F* value		2.5	7.9	8.2	.1
*P* value		n.s.	**.005**	.**004**	n.s.

**Table 4:** Correlations between cool down reactions, work engagement, and life satisfaction.

	Cool down index	Perceived work burden	Utrecht work engagement sum scores	Life satisfaction
Cool down reactions (CDI)		.248^**^	−.255^**^	**−.310** ^**^
Perceived work burden (VAS)			−.149^**^	−.239^**^
Work engagement—sum (UWES)	−.255^**^	−.149^**^		**.345** ^**^
Vigor	**−.319** ^**^	−.191^**^		**.393** ^**^
Dedication	−.261^**^	−.120^**^		**.307** ^**^
Absorption	−.161^**^	−.105		.250^**^
Aspects of spirituality (ASP)				
*Religious orientation *	−.078	−.044	**.368** ^**^	.151^**^
*Search for insight/wisdom *	−.107	−.036	**.330** ^**^	.137^**^
*Conscious interactions *	−.145^**^	−.165^**^	.256^**^	.234^**^
*Transcendence conviction *	−.106	−.044	.204^**^	.011

^**^ *P* < .01 (Spearman Rho).

Moderate correlations were highlighted (bold).

**Table 5:** Regression analyses with cool down reactions and life satisfaction as dependent variables (stepwise method).

		Beta	*T*	*P*	Collinearity statistics^*^	
					Tolerance	VIF
Cool down reactions (CDI)						
Model 5 (*R* ^2^ = .20)	(Constant)		8.879	.000		
	Life satisfaction (BMLSS-10)	−.195	−3.710	.000	.787	1.270
	Work burden (VAS)	.205	4.218	.000	.925	1.081
	Vigor (UWES)	−.221	−4.043	.000	.732	1.367
	Alcohol consumption	.183	3.831	.000	.951	1.052
	*Religious orientation *(ASP)	.108	2.109	.036	.835	1.198

Life satisfaction (BMLSS)						
Model 7 (*R* ^2^ = .35)	(Constant)		9.529	.000		
	Vigor (UWES)	.383	8.063	.000	.785	1.274
	Physicians versus others	.269	6.198	.000	.944	1.059
	Cool down reactions (CDI)	−.169	−3.709	.000	.857	1.167
	*Conscious interactions* (ASP)	.169	3.788	.000	.893	1.120
	Work burden (VAS)	−.138	−3.102	.002	.899	1.112
	Living with/without partner	.115	2.655	.008	.954	1.048
	*Transcendence conviction* (ASP)	−.103	−2.313	.021	.904	1.106

The following variables were not among the significant predictors of the respective models: gender, age, high school education, duration of years working on the respective profession, absorption, dedication, or *Search for insight/wisdom*.

^*^Because the regression coefficients may be compromised by collinearity, we checked the variance inflation factor (VIF) as an indicator for collinearity. VIF > 10 is indicative of high collinearity.

## References

[1] Felton J. S. (1998). Burnout as a clinical entity—its importance in health care workers. *Occupational Medicine*.

[2] McNeely E. (2005). The consequences of job stress for nurses' health: time for a check-up. *Nursing Outlook*.

[3] Adelman R. D., Tmanova L. L., Delgado D., Dion S., Lachs M. S. (2014). Caregiver burden: a clinical review. *Journal of the American Medical Association*.

[4] Halpern J. (2011). *From Detached Concern to Empathy: Humanizing Medical Practice*.

[5] Humphries N., Morgan K., Conry M. C., McGowan Y., Montgomery A., McGee H. (2014). Quality of care and health professional burnout: narrative literature review. *International Journal of Health Care Quality Assurance*.

[6] Biederbeck M., Gostomzyk J. G., Enke M. C. (2007). Belastungen in den Pflegeberufen und Möglichkeiten der Prävention. *Menschen für Gesundheit. Die Gesundheitsberufe*.

[7] Chou L.-P., Li C.-Y., Hu S. C. (2014). Job stress and burnout in hospital employees: comparisons of different medical professions in a regional hospital in Taiwan. *BMJ Open*.

[8] Ahrweiler F., Neumann M., Goldblatt H., Hahn E. G., Scheffer C. (2014). Determinants of physician empathy during medical education: hypothetical conclusions from an exploratory qualitative survey of practicing physicians. *BMC Medical Education*.

[9] Lee F. J., Stewart M., Brown J. B. (2008). Stress, burnout, and strategies for reducing them. What's the situation among Canadian family physicians?. *Canadian Family Physician*.

[10] Linzer M., Manwell L. B., Williams E. S. (2009). Working conditions in primary care: physician reactions and care quality. *Annals of Internal Medicine*.

[11] Rabin S., Shorer Y., Nadav M., Guez J., Hertzanu M., Shiber A. (2011). Burnout among general hospital mental health professionals and the salutogenic approach. *Israel Journal of Psychiatry and Related Sciences*.

[12] Pranjić N. (2012). Burnout and predictors for Burnout among physicians in Bosnia and Herzegovina-survey and study. *Acta Medica Academica*.

[13] Maslach C., Jackson S. E., Leiter M. P. (1996). *Maslach Burnout Inventory Manual*.

[14] McManus I. C., Winder B. C., Gordon D. (2002). The causal links between stress and burnout in a longitudinal study of UK doctors. *The Lancet*.

[15] Bakker A. B., Demerouti E., Schaufeli W. B. (2002). Validation of the Maslach burnout inventory-general survey: an internet study. *Anxiety, Stress and Coping*.

[16] Koenig H. G., King D., Carson V. B. (2012). *Handbook of Religion and Health*.

[17] Cobb M., Puchalski C. M., Rumbold B. (2012). *Oxford Textbook of Spirituality in Healthcare*.

[18] Zwack J., Schweitzer J. (2013). If every fifth physician is affected by burnout, what about the other four? Resilience strategies of experienced physicians. *Academic Medicine*.

[19] Anandarajah G., Roseman J. L. A. (2013). A qualitative study of physicians' views on compassionate patient care and spirituality: medicine as a spiritual practice?. *Rhode Island Medical Journal*.

[20] Doolittle B. R., M Windish D., Seelig C. B. (2013). Burnout, coping, and spirituality among internal medicine resident physicians. *The Journal of Graduate Medical Education*.

[21] Curlin F. A., Lantos J. D. (2005). Religious characteristics of U.S. physicians: a National Survey. *Journal of General Internal Medicine*.

[22] Ecklund E. H., Cadge W., Gage E. A., Catlin E. A. (2007). The religious and spiritual beliefs and practices of academic pediatric oncologists in the United States. *Journal of Pediatric Hematology/Oncology*.

[23] Cheever K. H., Jubilan B., Dailey T. (2005). Surgeons and the spirit: a study on the relationship of religiosity to clinical practice. *Journal of Religion and Health*.

[24] Lee E., Baumann K. (2013). German psychiatrists' observation and interpretation of religiosity/spirituality. *Evidence-Based Complementary and Alternative Medicine*.

[25] Glöckler M., Glöckler M. (2014). Grundlagen der anthroposophischen medizin. *Anthroposophische Arzneitherapie für Ärzte und Apotheker*.

[26] Kienle G. S., Albonico H. U., Baars E., Hamre H., Zimmermann P., Kiene H. (2013). Anthroposophic medicine: an integrative medical system originating. *Global Advances in Health and Medicine*.

[27] Heusser P. (2011). *Anthroposophische Medizin und Wissenschaft: Beiträge zu Einer Ganzheitlichen Medizinischen Anthropologie*.

[28] Steiner R. (1994). *How to Know Higher Worlds. A Modern Path to Initiation*.

[29] Glöckler M. (2013). Meditativer erkenntnisweg und selbstschulung in der anthroposophischen medizin. *Der Merkurstab*.

[30] Steiner R., Wegman I. (1983). *Fundamentals of Therapy. An Extension of the Art of Healing through Spiritual Knowledge*.

[31] Steiner R. (1997). *Course for Young Doctors: Meditative Contemplations and Instructions for Deepening the Art of Healing*.

[32] Girke M. (2012). *Innere Medizin: Grundlagen und Therapeutische Konzepte der Anthroposophischen Medizin*.

[33] Glöckler M., Glöckler M. (2002). Wie lässt sich Menschlichkeit lernen?. *Spirituelle Ethik*.

[34] Müller A. (2012). *Spiritualität, Lebenszufriedenheit und Cool down in der Alten-und Krankenpflege [Diploma thesis]*.

[35] Schaufeli W. B., Salanova M., González-Romá V., Bakker A. B. (2002). The measurement of engagement and burnout: a two sample confirmatory factor analytic approach. *Journal of Happiness Studies*.

[36] Schaufeli W. B., Taris T. W., Van Rhenen W. (2008). Workaholism, burnout, and work engagement: three of a kind or three different kinds of employee well-being?. *Applied Psychology*.

[37] Büssing A., Fischer J., Haller A., Heusser P., Ostermann T., Matthiessen P. F. (2009). Validation of the brief multidimensional life satisfaction scale in patients with chronic diseases. *European Journal of Medical Research*.

[38] Büssing A., Ostermann T., Matthiessen P. F. (2007). Distinct expressions of vital spirituality: the ASP questionnaire as an explorative research tool. *Journal of Religion and Health*.

[39] Büssing A., Kerksieck P., Föller-Mancini A., Baumann K. (2012). Aspects of spirituality and ideals to help in adolescents from christian academic high schools. *International Journal of Children's Spirituality*.

[40] Büssing A., Hirdes A. T., Baumann K., Hvidt N. C., Heusser P. (2013). Aspects of spirituality in medical doctors and their relation to specific views of illness and dealing with their patients' individual situation. *Evidence-Based Complementary and Alternative Medicine*.

